# Overcoming genetic and cellular complexity to study the pathophysiology of X-linked intellectual disabilities

**DOI:** 10.1186/s11689-024-09517-0

**Published:** 2024-02-29

**Authors:** Dayne Martinez, Evan Jiang, Zhaolan Zhou

**Affiliations:** 1 Department of Genetics, University of Pennsylvania Perelman School of Medicine, Philadelphia, PA 19102 USA; 2 Medical Scientist Training Program, University of Pennsylvania Perelman School of Medicine, Philadelphia, PA 19102 USA; 3 Department of Neuroscience, University of Pennsylvania Perelman School of Medicine, Philadelphia, PA 19102 USA; 4 Intellectual and Developmental Disabilities Research Center, Children’s Hospital of Philadelphia, Philadelphia, PA 19104 USA

## Abstract

X-linked genetic causes of intellectual disability (ID) account for a substantial proportion of cases and remain poorly understood, in part due to the heterogeneous expression of X-linked genes in females. This is because most genes on the X chromosome are subject to random X chromosome inactivation (XCI) during early embryonic development, which results in a mosaic pattern of gene expression for a given X-linked mutant allele. This mosaic expression produces substantial complexity, especially when attempting to study the already complicated neural circuits that underly behavior, thus impeding the understanding of disease-related pathophysiology and the development of therapeutics. Here, we review a few selected X-linked forms of ID that predominantly affect heterozygous females and the current obstacles for developing effective therapies for such disorders. We also propose a genetic strategy to overcome the complexity presented by mosaicism in heterozygous females and highlight specific tools for studying synaptic and circuit mechanisms, many of which could be shared across multiple forms of intellectual disability.

## Background

Intellectual disability (ID) represents one of the leading causes of disability worldwide and is characterized by impaired general mental functioning linked with deficits in conceptual, social, and practical skills. The presentation of ID is highly heterogeneous, occurring on a spectrum of severity and in a variety of contexts including ID alone, ID comorbid with another neurodevelopmental disorder (NDD), or ID as part of a syndrome [1]. Its prevalence globally is estimated to be 1–3% of the population [2].

Nongenetic factors such as fetal alcohol exposure, environmental toxins, infectious agents, and injuries are a major cause of ID, accounting for roughly half of all cases with an identifiable cause [3]. Genetic etiologies are estimated to account for another half of ID cases [3–5] and include chromosomal abnormalities, disruptions to individual genes (i.e., monogenic), deleterious interactions between multiple genes (i.e., polygenic), imprinting disorders, and mitochondrial genetic disorders [6]. This review focuses on X-linked forms of ID that result from disruption of genes carried on the X chromosome. Out of almost 1400 genes associated with IDs, over 140 are X-linked [5, 7, 8]. The research and treatment of these disorders are often hindered and limited by the additional complexity introduced by the unique biology of the X chromosome.

### The unique biology of X-linked IDs is complex and understudied

In most mammals, female offspring inherit both a maternal and paternal X chromosome, while males inherit a maternal X chromosome and a paternal Y chromosome. Cells in early female embryos silence one of their two X chromosomes through epigenetic mechanisms to prevent the overproduction of gene products, such that cells in both males and females ultimately have only one active X chromosome. In placental mammals, including humans and commonly used murine models, this silencing occurs randomly with an equal chance that the maternal or paternal X chromosome is inactivated. The silenced chromosome remains inactive in the cell and its descendants for the lifetime of the organism [9, 10]. This phenomenon, known as X chromosome inactivation (XCI), has been an interesting focus of investigation since it was first proposed by Mary Lyon in 1961 [11], and it has substantial implications for the research and treatment of X-linked disorders.

Because they possess only one X chromosome, hemizygous males usually have more severe clinical symptoms than heterozygous females carrying similar mutations in an X-linked gene. Having a second X chromosome carrying a functional copy of a gene allows heterozygous females to compensate for a mutation; however, the mechanisms and efficacy of this compensation are not straightforward due to XCI. Apart from a minority of genes that escape XCI, cells in a heterozygous female will express either the functional copy or the pathogenic copy of a gene, but not both. This leads to a mosaic expression pattern despite the cellular genotypes being the same. For this review, the terms “mosaic” and “mosaicism” refer to a pattern of gene expression among a population of cells. A situation where cells within an organism have a difference in genotype will be specified as somatic mosaicism [12].

Mosaic females may be able to compensate in part by either maintaining tissue function if a sufficient proportion of cells express the wild-type copy or if mutant-expressing cells are able to obtain missing gene products from their wild-type neighbors via processes like intercellular transfer through gap junctions or endocytosis. Furthermore, an individual may show skewed XCI, a situation where one chromosome is disproportionally inactivated, due to chance or factors affecting the proliferation and survival of cells expressing one of the chromosomes. A heavy skew in favor of the wild-type chromosome could be protective, whereas a bias toward the mutant could be harmful. A skew can also be localized to a particular tissue or anatomical region, which contributes to the broader range of phenotypic severity that is often observed in females [12].

While most genes on the X chromosome are subject to XCI, a minority of genes on the inactivated X chromosome escape XCI and remain expressed or partially expressed, which presents another layer of complexity that must be considered when studying X-linked genetic disorders in heterozygous females. There are several models that attempt to explain the escape of XCI by considering the different evolutionary pathways that could contribute to the emergence and specialization of modern sex chromosomes [13]. Escape from XCI appears to be a tightly controlled process in mice and humans that is dependent on tissue and cell type. In mice, an estimated 3–7% of genes escape XCI [14]. In humans, the proportion is about 15–30%, depending on the tissue and cell type [15–17]. Most genes associated with X-linked disorders are subject to XCI; however, several genes that show escape have been linked to NDDs (*DDX3X*, *IQSEC2*, and *KDM6A* among others) [18].

The heterogeneity that XCI generates has confounded attempts to study the pathological mechanisms of X-linked disorders, especially those with a substantial number of heterozygous female patients. In the case of X-linked forms of ID, this challenge is compounded greatly by the complexity of the brain and the circuits that underly mental functions.

In the next few sections, we will provide a brief overview of several monogenic forms of syndromic ID that manifest predominantly in heterozygous females. While the genetic etiologies of these disorders are distinct, they share many features with each other and other NDDs at the level of cellular and behavioral phenotypes. The hope is that studying these disorders will yield not only a better understanding of the individual disorders but also to produce insights in neurobiology applicable to ID and other NDDs more generally. The final sections consist of a discussion of how therapeutic strategies are evolving to overcome the complexity introduced by mosaicism as well as a proposal of novel experimental strategies for studying synapse and circuit biology in mosaic models of X-linked disorders.

### Rett syndrome

Rett syndrome (RTT) is a severe developmental encephalopathy caused by mutations in the X-linked *MECP2* gene [19] and represents one of the most common genetic causes of ID in females with an estimated incidence of 1 per 10,000 female births [20]. The vast majority of RTT patients are heterozygous females. Male RTT patients are exceedingly rare, both because mutations in *MECP2* are more likely to be *de novo* in paternal gametes and because lacking any functional copies of *MECP2* results in early lethality in males. Co-occurrence of Klinefelter syndrome (where males carry two X chromosomes and one Y chromosome), somatic mosaicism, or less disruptive mutations is usually involved in male cases of RTT [21]. RTT has a classic clinical progression; however, the disease can present differently depending on a variety of factors, including but not limited to sex, degree of mosaicism (due to XCI or somatic mosaicism), and the specific mutation involved [20].

Classic RTT has a characteristic progression from a period of apparently normal development for about 6–18 months to a period of stalled development and marked regression where motor and social skills that had been acquired are lost and microcephaly is commonly observed. The capacity for purposeful hand movement deteriorates and is replaced by hand stereotypies such as clapping or wringing, and many children also develop ataxia or tremors. Social withdrawal, respiratory complications due to autonomic dysfunction, and sleep disturbances also develop during this stage. Later in childhood, motor and social skills stabilize and may partially recover; however, cognitive impairments are apparent, and seizures can develop during this period. After the first decade of life, patients may show improvement in cognitive, social, and emotional skills and a reduced frequency of seizures; however, they can also develop other problems such as parkinsonism (rigidity, bradykinesia, and tremor), scoliosis, and osteoporosis [22].

The molecular, cellular, and circuit mechanisms that underly RTT remain to be fully understood. *MECP2* encodes methyl-CpG-binding-protein 2 (MeCP2), an abundant nuclear protein that interacts broadly with chromatin and specifically binds methylated DNA [23]. MeCP2 is expressed in most cell types of the body, but it is highly enriched in neurons [24]. Early studies of MeCP2 function *in vitro* suggested a role as a repressor of gene expression [25–27]; however, later studies using next-generation sequencing revealed that while the transcription of many genes increases upon the loss of MeCP2, other genes show a decrease in transcription [28–33]. The changes in gene expression are broad with hundreds of differentially expressed genes identified in models of RTT, and the changes are generally small in magnitude, which suggests that MeCP2 does not function as a classical repressor, but rather that MeCP2 is important for tuning the transcription of genes more globally [34].

**Fig. 1:**
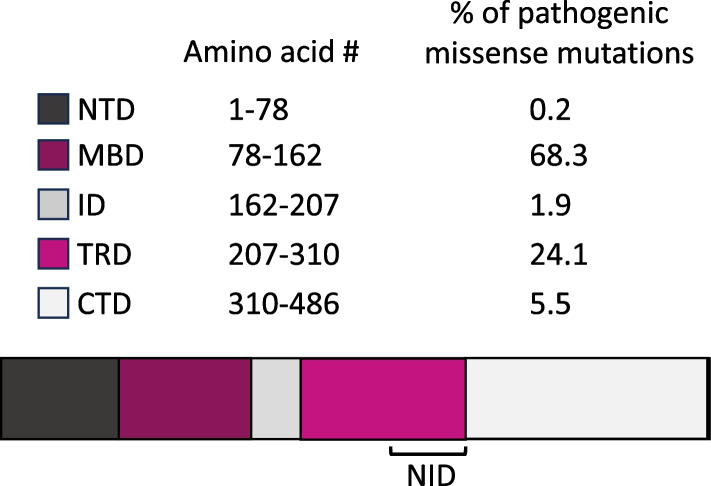
Distribution of pathogenic missense mutations within MeCP2. Diagram of the MeCP2 protein with its major domains. The percentage of pathogenic missense mutations that map to each domain is given and was adapted from previously reported analysis of multiple clinical databases [37]. Abbreviations: NTD, N-terminal domain; MBD, methyl-DNA-binding domain; ID, interdomain; TRD, transcription repressor-binding domain; NID, NCoR/SMRT interacting domain; CTD, C-terminal domain

The molecular mechanisms by which MeCP2 regulates transcription have been a subject of active investigation, though the genetic data cataloging the sites of missense mutations in RTT patients suggests that the binding of MeCP2 to chromatin and its interaction with the NCoR-SMRT complex are critical. These pathogenic mutations cluster in the methyl-CpG-binding domain (MBD) that enables MeCP2 binding to methylated DNA and the NCoR-SMRT interacting domain (NID) that allows MeCP2 to bind to the NCoR-SMRT co-repressor complex (Fig. 1), suggesting that one important role of MeCP2 is acting as a bridge between methylated DNA and transcriptional regulation machinery [35]. In addition to a role regulating transcription, MeCP2 has also been suggested to influence chromatin architecture [34, 36].

MeCP2 has been shown to bind to methylated cytosines in both the CG and CH context (where H represents an A, T, or C nucleotide, named as mCG and mCH hereafter), with mCA being more common than mCT and mCC in the mammalian brain. MeCP2 also shows high affinity for hydroxylated mCA (hmCA), but not hmCG, based on *in vitro* binding assays, though detectable hmCA *in vivo* in the brain is rare [32, 38]. In contrast, hmCG shows a significant increase alongside mCA during early postnatal development and reaches uniquely high levels in postmitotic neurons. The accumulation of hmCG and mCA is specific to neurons and is correlated with changes in gene expression, suggesting that these forms of DNA methylation could have important roles in the maturation and function of neurons [24, 39–43]. Thus, changes in the DNA methylation landscape over the course of development likely dictates the genome-wide occupancy of MeCP2 and impact its molecular function in the brain.

It remains unclear how the disruption of a DNA methylation-binding protein that is present in every tissue and cell type produces the neurologic deficits observed in RTT. Mouse models carrying knockout [44, 45] and knockin [30, 35, 46–51] mutations of MeCP2 have been generated and recapitulate multiple features of RTT. Even though most of the patient population are heterozygous females, mechanistic studies have often been limited to hemizygous male mice, largely to avoid the confounding effects of random XCI. A few studies that included heterozygous females identified changes in synapse and dendrite morphology [52, 53], synaptic functioning [54], and behavioral phenotypes [44], which supports the idea that MeCP2 has important roles in the CNS. Further studies will be needed to understand the contributions of mosaicism and non-cell-autonomous effects toward synapse and circuit functioning and RTT pathophysiology more generally.

### CDKL5 deficiency disorder

CDKL5 deficiency disorder (CDD) is a debilitating childhood disorder and one of the most common forms of genetic epilepsy with an estimated incidence of 1 in 40,000–60,000 live births [55]. A cardinal symptom of CDD is early-onset seizures that emerge within the first few months of life and are mostly refractory to anti-epileptic medications. Other neurologic features of CDD include intellectual disability, motor and visual impairments, sleep disturbances, and autistic features [56–58]. CDD is caused by loss-of-function mutations in the X-linked cyclin-dependent kinase-like 5 (*CDKL5*) gene [59–63], a serine-threonine kinase expressed highly in neurons of the brain [64–66].

**Fig. 2:**
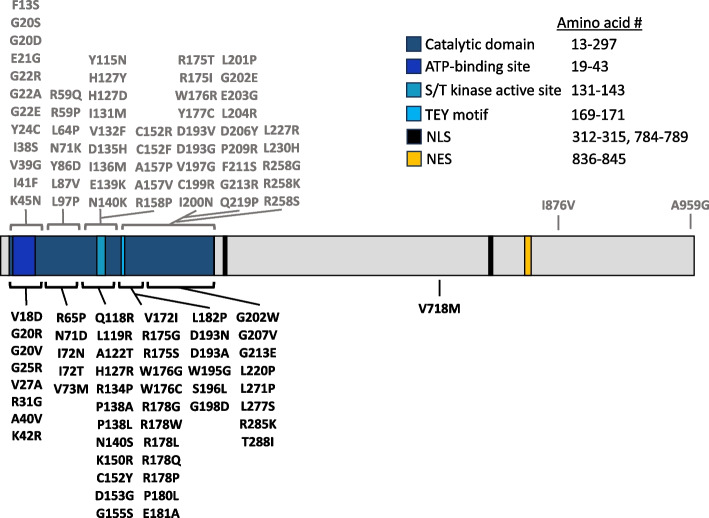
Pathogenic missense mutations in CDKL5 map to the catalytic domain. Schematic of the human CDKL5 protein isoform predominant in the brain. Missense mutations classified as pathogenic by the ClinVar [70] database are mapped beneath the protein in black, while those categorized as likely pathogenic are noted above in gray. Abbreviations: NLS, putative nuclear localization signal; NES, putative nuclear export signal

CDD predominantly affects heterozygous females with a 4:1 female-to-male ratio [56]. Males typically have a more severe phenotype than females. One study suggests that this difference is not necessarily large on average, however data interpretation is complicated by the presence of somatic mosaicism in a proportion of male patients [67]. Missense mutations cluster in the kinase domain of the CDKL5 protein (Fig. 2), emphasizing the importance of the signaling function of CDKL5 [68]. Attempts to correlate specific variants with phenotypic severity have yielded limited insights and are hindered by the variability that follows random XCI [69].

Given that CDKL5 is a serine-threonine kinase, several studies have investigated changes to signaling pathways in models of CDD [71, 72]. Chemical genetic and phosphoproteomic screens to identify substrates of CDKL5 identified cytoskeleton-associated proteins EB2, MAP1S, and ARHGEF2 as putative substrates, suggesting a role regulating the cytoskeleton and associated cellular functions [73, 74]. Indeed, CDKL5 appears to be important for the proper functioning of cilia [75]. CDKL5 has also been reported to be present in the nucleus [76] and to have a role in regulating the DNA damage response, possibly through phosphorylation of the transcriptional regulator ELOA [77].

Accumulating evidence supports that CDKL5 localizes to and regulates the functioning of synapses. CDKL5 has been shown to interact with the postsynaptic density scaffolding protein PSD-95 in a manner dependent on PSD-95 palmitoylation and the large intrinsically disordered C-terminus of CDKL5 [66]. CDKL5 has also been shown to interact with the postsynaptic adhesion protein NGL-1 [65]. One study reported that CDKL5 was able to phosphorylate AMPH1, a synapse-enriched protein that participates in synaptic vesicle recycling [78]. A separate study demonstrated that CDKL5 is present at the presynaptic terminal, and that CDKL5 KO neurons have deficits in synaptic vesicle recycling, though this was not related to AMPH1 phosphorylation status [79]. Glutamatergic signaling is also altered upon loss of CDKL5, with two reports of an increase in synaptic NMDAR subunit GluN2B in CDKL5 KO mice [80, 81]. Multiple studies have described changes in dendrite morphology and spine number and morphology [65, 82–85], though the nature and reproducibility of these findings have varied. The discrepancies may be attributable to comparing *in vitro* versus *in vivo* systems, utilizing different models, and a focus on different brain regions, cell types, or ages.

Patient derived iPSCs have been generated, enabling the study of disease-related mutations of CDKL5 in human neurons, including cerebral organoids [72, 86]. Most *in vivo* studies have been performed using mouse models, with several CDKL5 KO lines being described in the literature [71, 80, 81, 84, 87]. These mouse models have been able to recapitulate multiple features of CDD, including learning and memory impairments and social deficits [71, 88, 89]. None of the mouse models have reported spontaneous seizures in mice with complete loss of CDKL5, though abnormal evoked event-related potentials (ERPs) and altered drug-induced seizure threshold have been observed [71, 80, 84].

Interestingly, seizures are observed in heterozygous female mice from multiple lines upon aging [90, 91]. Given that seizures are not observed in hemizygous male or homozygous female KO mice and that the average age of seizure development in heterozygous mice is around 28 weeks of age, it is unlikely that the seizures observed in heterozygous mice reflect epileptogenic processes identical to those present in neonatal humans. Rather, mosaic expression of CDKL5 following random X-chromosome inactivation could exacerbate the underlying predisposition to circuit hyperexcitability found in models of CDD. Another possibility is that total loss of CDKL5 induces a more robust compensatory response early in development that prevents the emergence of seizures later in mice. It could also be possible that heterozygous female patients experience age-dependent changes that have not been documented. While it is unclear why seizures are specifically observed in heterozygous mice, a substantial majority of CDD patients are heterozygous females, and a smaller number are somatic mosaic males and females [58], which suggests that identifying processes that could amplify circuit hyperexcitability in mosaics could be directly relevant to the majority of CDD cases.

### PCDH19 epilepsy

PCDH19 epilepsy has an estimated incidence of about 1 per 21,000 live births [55] and is caused by mutations in the *PCDH19* gene, which encodes a protocadherin belonging to the cadherin superfamily of cell-adhesion proteins [92, 93]. Patients usually begin exhibiting clusters of febrile and afebrile seizures before 3 years of age, usually within the first several months of life. The seizures are typically refractory to treatment with anti-epileptic medications, though seizures tend to become less frequent after childhood. Severe cognitive impairment is rare, with most patients displaying mild to moderate impairment and many other patients having scores consistent with normal cognitive functioning. Autistic features and behavioral dysregulation are common in PCDH19-epilepsy patients [94–96].

A distinguishing aspect of PCDH19 epilepsy is that it presents in heterozygous females but not in hemizygous males, with males instead generally showing normal neurologic functioning. Interestingly, males who are somatic mosaic carriers of *PCDH19* mutations are affected similarly to heterozygous females. Several mechanisms have been proposed to contribute to the pathophysiology of PCDH19 epilepsy including GABA receptor dysregulation [97], blood-brain barrier dysfunction [98], impaired steroid metabolism [99], asynchronous neurogenesis [100], and cellular interference [93]. Of these, the human genetic and animal model data suggest that cellular interference, which is the concept that cells expressing different alleles of a gene have deleterious interactions with each other but not with cells expressing the same allele, is the primary mechanism underlying PCDH19-epilepsy pathophysiology.

Cellular interference is a term that was first used to describe EFNB1 syndrome, a genetic disorder caused by mutations in the *EFNB1* gene that is characterized by craniofacial deformations and has the same inheritance pattern as PCDH19 epilepsy, with heterozygous females and mosaic males being most severely affected [101–103]. Cellular adhesion molecules, including protocadherins, are necessary for proper neuronal migration and participate in transsynaptic connectivity as well as signaling pathways that are critical for the development, specification, and functioning of neural circuits [104–106]. In animal models that are heterozygous for PCDH19 mutations, neurons show abnormal cellular sorting, with neurons carrying the same allele of PCDH19 clustering together and forming synapses with each other but not with neurons carrying the other variant, leading to abnormal neural circuit formation. However, this abnormal cellular sorting does not occur in animals that only carry the mutant PCDH19, such as hemizygous males, which leads to relatively normal neural circuit development [107]. Furthermore, neurons expressing different alleles of PCDH19 appear to have dysregulated transsynaptic signaling due to the incompatibility of the synaptic adhesion protein complexes formed by neurons that have functional PCDH19 with the different complexes that form in neurons that do not, which leads to impaired synapse development and functioning [108].

PCDH19 epilepsy is a dramatic example of the principle of cellular interference, and while most other X-linked disorders do not share the same inheritance pattern, it does raise the possibility that mosaicism can lead to intercellular signaling incompatibilities or other problems in other X-linked disorders. While the net effect of mosaicism is usually positive, that does not preclude the possibility that there are also deleterious processes that are occurring alongside beneficial ones in the context of heterozygous females and somatic mosaics. However, this is a largely unexplored possibility, in part because investigating such possibilities is technically challenging.

### X-linked IDs provide unique challenges and opportunities for therapeutic development

A conceptually direct way to treat monogenic NDDs is to replace or supplement the gene or gene product that is disrupted. Indeed, genetic rescue studies using reversible KO alleles in animal models of multiple NDDs support the theoretical efficacy of this approach [84, 109, 110]. In human patients, virus-mediated gene therapy is one of the primary strategies that are being developed to this end.

AAV has been the most popular vehicle of choice thus far for *in vivo* gene therapies for several reasons. There are multiple serotypes of AAV that naturally have tropism for different tissues, with AAV9 being the standard serotype for CNS gene therapies due to its ability to cross the blood-brain barrier and its tropism for neurons and glial cells, though there is an ongoing effort to engineer novel AAV variants to expand and improve upon available tools. The viral DNA carried by recombinant AAV can be limited to inverted terminal repeats (ITRs) that flank the expression cassette, which itself usually consists of a promoter, the gene to be expressed, a polyA tail, and possible other regulatory elements. Furthermore, the viral DNA from AAV can circularize to form episomes that can be expressed and persist in eukaryotic cells with minimum integration into the host genome, which avoids the potential oncogenic risks of viruses that mediate genomic integration of transgenes, such as retroviruses. On the other hand, use of AAV for gene therapy also has several limitations. Namely, they have a limited packaging capacity, the transduction efficiency in the CNS is relatively limited, and the treatment can potentially trigger a severe immune response. Additionally, there are potential toxic effects from overexpression or off-target expression of a transgene [111].

The potential toxicity of overexpressing a gene of interest is especially relevant for X-linked disorders that present in heterozygous females and somatic mosaics because a substantial proportion of their cells are already expressing a functioning copy of the gene. As discussed previously, mutations in *MECP2* lead to Rett syndrome. However, duplication of *MECP2* also has substantial neurological consequences. While there are differences in the details when comparing the disorders, *MECP2* duplication syndrome is a severe developmental encephalopathy characterized by many of the same deficits that affect RTT patients [22]. This suggests that expressing too much MeCP2 protein in cells that are already expressing a functional copy could be deleterious and potentially counter the benefits of introducing MeCP2 in mutant-expressing cells. Furthermore, it raises the possibility that other X-linked genes may be similarly sensitive to dosage, with too little or too much gene product being toxic.

Because of the additional risk of toxicity from overexpressing a gene in mosaic patients, adjusted and alternative gene replacement strategies are being developed. One approach in RTT models has been to modify the transgene to destabilize the RNA and reduce protein translation efficiency, which reduces the overall amount of MeCP2 that is produced [112]. A different method to reduce potential overexpression toxicity utilizes additional regulatory elements, such as microRNA, to moderate transgene expression [113]. These approaches try to allow for a high enough dosage of treatment to affect as many cells as possible while also not overloading any given cell with too much gene product, but the best way to strike a balance has yet to be determined.

Notably, all the above strategies to mitigate toxicity from overexpressing gene product lack specificity. In other words, they introduce a transgene or gene product into all cells regardless of which X chromosome is active (and therefore regardless of which allele of the X-linked gene is expressed). One possible solution to eliminate the possibility of toxic overexpression more precisely is X-chromosome reactivation [114]. The goal of this approach is to partially reactivate the X chromosome to allow functional copies of the gene of interest to be expressed in cells that had silenced them previously through XCI. A few studies have developed pharmacologic methods to induce escape from XCI [115–117]. Others have attempted to leverage the specificity of CRISPR-mediated targeting by using catalytically inactive dCas9 to bring a DNA methylation modifying enzyme, TET, and transcriptional activating machinery specifically to the gene of interest [118–120]. Even though this strategy has theoretical promise given that it would introduce a single functioning copy of the gene of interest in each cell, there are still several limitations. The methods for efficiently enabling the expression of the gene of interest specifically need substantial improvement. Furthermore, this approach will not be effective if the mutant allele of the gene has dominant negative effects, and it is not applicable in somatic mosaic males, which do not have a second, functional copy of a gene to express given their singular X chromosome.

On the other hand, the synapse has emerged as a common node that could be targeted to treat multiple forms of ID. Large-scale GWAS and WES/WGS studies converge on genes important for synaptic function, suggesting synapse dysregulation as a major contributor to many NDDs including ID [121]. It is common for synapses in different IDs and other NDDs to show deficits in structure and function, many of which are similar across disorders. This suggests that targeting key signaling pathways that regulate synapse development, plasticity, and functioning could be a way to develop therapeutics that can be applied to multiple disorders rather than a single, specific disorder.

It must be noted, however, that synaptic interactions are more complex in mosaic females. When neurons (and glia) interact to form synapses and circuits, the interaction can be between cells expressing the same allele of a gene (termed *homoallelic*) or cells expressing different alleles (termed *heteroallelic*). Furthermore, synapses are directional, with differentiated pre- and postsynaptic structures. This presents a situation where there could be symmetric or asymmetric changes on either side of a synapse, with the possibility that different combinations have different consequences for synapse formation and functioning. PCDH19 epilepsy is a dramatic example where mismatched synaptic interactions have deleterious consequences [96], but the possibility of non-cell-autonomous effects in other disorders, such as the observation in models of RTT that even wild-type neurons can show synaptic deficits if the synapse is interacting with a MeCP2 null astrocyte [54], suggest that these specific interactions could be making unappreciated contributions to the pathophysiology of similar disorders. Identifying and studying these kinds of interactions in mosaic models and patients will contribute to a better understanding of disease pathophysiology and open new avenues for potential therapeutic intervention.

### Novel tools and genetic strategies to account for random XCI in the heterozygous female brain

Overcoming mosaicism to study X-linked disorders is challenging. Previously generated mouse models [30] and single-cell transcriptomic profiling methods [122] enable characterization at the level of individual cells; however, resolving the striking heterogeneity of synapses and circuits in mosaics is beyond the scope of these previous approaches. Given the time and resources required to generate new model systems and sophisticated bioinformatic pipelines, devising simpler strategies for overcoming mosaicism that can be applied more broadly would accelerate the ability of researchers studying X-linked disorders that present in mosaics to investigate disease mechanisms thoroughly in models that better represent the patient population of interest (rather than relying on hemizygous male models for to avoid confounds from mosaicism). To this end, we propose a strategy that uses X-linked genetic tools and an F1 cross-breeding scheme as a generalizable method for overcoming mosaicism in models of X-linked disorders, and we provide a specific illustration of how this might be used to study synapse and circuit function in mosaics with synapse-level resolution.

One previous study of mosaicism in the heterozygous female mouse brain made use of nuclear-localized fluorescent reporters that were inserted upstream of the X-linked *HPRT1* gene [123]. This strategy involved crossing mice with an X-linked green reporter with mice carrying an X-linked red reporter. In F1 heterozygous female offspring, a cell would express either the green or the red reporter, but not both, following random XCI. These genetic constructs exhibited strong reporter expression in a pattern indicating little to no detectable escape from XCI. This suggests that this strategy could be adapted to reliably express additional reporters and tools in an X-linked fashion using transgenes inserted upstream of the *HPRT1* locus (and potentially other X-linked genes), which could provide a generalizable means of overcoming heterogeneity in mosaics.

For example, if a mouse line carrying a green, X-linked fluorescent reporter is crossed with a mouse line carrying a mutant allele of a disease-related X-linked gene, the F1 heterozygous female offspring will have wild-type cells that can express the green reporter and mutant cells that cannot, assuming the gene does not escape XCI. This is because the chromosome carrying the reporter also carries the wild-type allele of every other X-linked gene. If the X chromosome carrying the mutant allele is inactivated, then the chromosome carrying the reporter and wild-type allele is expressed. Conversely, if the mutant chromosome is expressed, then the wild-type chromosome carrying the reporter will be silenced instead. The expression of the reporter is strictly linked to the expression of the wild-type allele in the somatic cells of the F1 offspring, which provides a reliable means of distinguishing wild-type and mutant cells in mosaic F1 females.

**Fig. 3:**
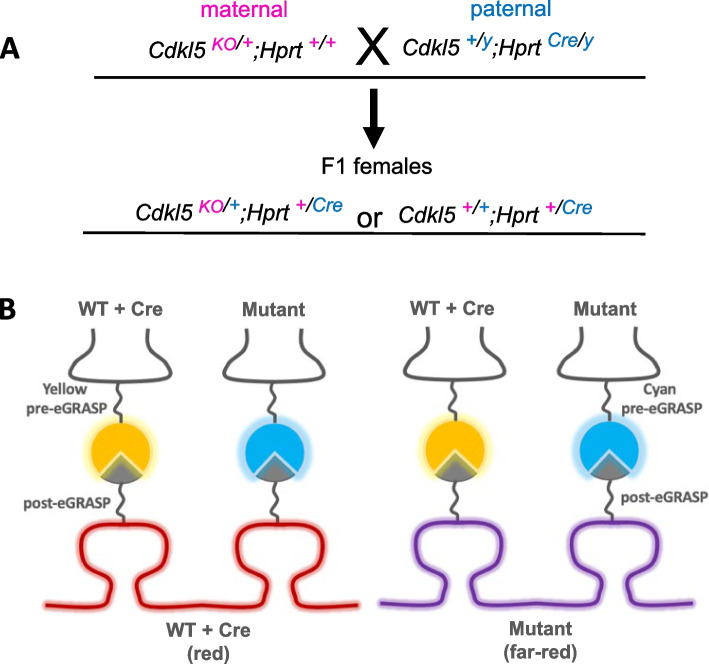
Genetic strategy to label homoallelic and heteroallelic synaptic interactions using dual-eGRASP. **A** Upon crossing wild-type male mice carrying an X-linked Cre (such as *Hprt*^*Cre*^ in blue text) to heterozygous female mice carrying a mutation in an X-linked gene of interest (such as *Cdkl5*^*KO*^ in pink text, as an example), Cre-expressing cells in the F1 female progenies will always express wild-type *Cdkl5*^*+*^, while *Cdkl5*^*KO*^-expressing cells will be Cre negative, due to the linkage of *Hprt*^*Cre*^ and *Cdkl5*^*+*^ on the same X-chromosome (WT with Cre, mutant without Cre). **B** When the dual-eGRASP system is delivered using Cre-ON and Cre-OFF AAV, specific synaptic interactions can be labeled in these F1 offspring. In this example, presynaptic cell populations are labeled using Cre-ON yellow pre-eGRASP and Cre-OFF cyan pre-eGRASP, while postsynaptic cells are labeled using Cre-ON membrane-bound red reporter with post-eGRASP and Cre-OFF membrane-bound far-red reporter with post-eGRASP

This approach is adaptable and can make use of several different tools. In addition to X-linked reporter lines, there are mouse lines that carry tamoxifen-inducible Cre recombinase (Cre-ER) at the same location upstream of *HPRT1* that was previously mentioned [124]. When mice carrying the X-linked Cre-ER are crossed with a disease model carrying a mutation on an X-linked gene, heterozygous F1 females will have Cre-ER activity restricted to cells expressing the wild-type allele, whereas cells expressing the X chromosome carrying the mutant allele will inactivate the chromosome carrying Cre-ER (Fig. 3A). The result is a mosaic where wild-type expressing cells possess Cre-ER and mutant expressing cells do not. When combined with other genetic tools for labeling and manipulating specific cell populations, such as additional mouse lines or AAV, this differential expression of Cre-ER provides a powerful method for studying a variety of biological processes in mosaic models. Given the prevalence of synaptic dysregulation in different forms of ID and other NDDs, the next section focuses on two different tools that can be adapted to study synapses and circuits while accounting for the complexity of mosaic expression of X-linked genes in heterozygous female models.

The first tool, dual-eGRASP, focuses on labeling and visualizing specific synaptic interactions. Early forms of GRASP (GFP reconstitution across synaptic partners) in mammalian brains allow for the visualization of synapses using the functional complementation of two nonfluorescent GFP fragments [125]. The fragments are expressed separately on the presynaptic and postsynaptic membrane and reconstitute in the synaptic cleft to form functional GFP. Dual-eGRASP (dual-enhanced GRASP) is an extension of this technique that enables visualization of synaptic interactions originating from two distinct pre- and postsynaptic cell populations by varying the color of the fluorescent proteins expressed by presynaptic and postsynaptic populations [126–128]. Key residues in the presynaptic GFP fragment were mutated to form new fragments that fluoresce yellow or cyan instead of green, but both can be complemented by the same postsynaptic GFP fragment. Different postsynaptic populations that express the smaller postsynaptic fragment can be labeled with distinct colors of membrane-bound reporters, such as red and far-red, to identify the cell population while also providing high definition of the structural features of the neuron and its synapses. When Cre-ON and Cre-OFF AAV carrying these dual-eGRASP constructs are combined with the F1 X-linked Cre strategy discussed above, it can enable the labeling of specific interactions in a way that is linked to the expression status of the X-linked gene of interest (Fig. 3B). This approach could enable studies of synapse density, morphology, and distribution, and it could be complemented by electrophysiology, imaging using calcium or voltage sensors, and optogenetic or chemogenetic tools to investigate the functional properties of synapses and circuits [129]. In this way, dual-eGRASP provides an elegant solution for studying synapse and circuit changes in X-linked ID and other NDDs, an area that has previously been difficult to approach due to random XCI.

Another tool for dissecting out specific synaptic interactions is split biotin ligases [130] and peroxidases [131]. Biotin ligases and peroxidases can biotinylate nearby proteins, which enables the proximity-based labeling and purification of those proteins using streptavidin [132, 133]. TurboID is a promiscuous biotin ligase that can label proteins quickly *in vivo* upon introduction of exogenous biotin [134]. Split TurboID is a version of this biotin ligase that has been divided into two fragments that lack function individually but can reconstitute to form a functional enzyme when in close proximity, which can be achieved in the synaptic cleft [135]. This system has been used previously to study the proteomic content of astrocyte-neuron interactions [136], suggesting that this system could be adapted for labeling and studying specific synaptic interactions in mosaics by combining an F1 X-linked Cre breeding strategy with Cre-ON and Cre-OFF AAV to deliver the split-TurboID fragments, similar to the dual-eGRASP strategy discussed above. In addition to labeling interactions *in situ*, the biotinylation of synaptic cleft proteins could also enable multi-omic profiling studies of specific synaptic interactions, either through direct purification of biotinylated proteins or potentially by generating synaptosomes and purifying labeled synapses using fluorescence-activated synaptosome sorting (FASS) [137, 138] or possibly streptavidin-mediated enrichment protocols similar to those for purifying organelles [139–142].

## Conclusion

Genetic causes of ID and related NDDs have complex pathophysiology and are notoriously difficult to study. Many of these disorders are X-linked, and the study and treatment of X-linked disorders that occur in heterozygous females have been especially challenging. This is due in part to the added heterogeneity and complexity following from mosaic expression of most X-linked genes after random XCI. Recent advances in gene therapy and the emergence of alternative or complementary approaches, such as selective X chromosome reactivation, are promising but face substantial technical obstacles that remain to be addressed. We have proposed a genetic strategy that makes use of X-linked reporters or Cre recombinase to overcome the heterogeneity of mosaic models of these X-linked disorders, which aims to facilitate a better understanding of the pathophysiology in systems that better reflect clinical populations. While progress has been challenging, the continuing evolution of research and therapeutic tools and strategies promises to open new avenues for therapeutic development.

## Data Availability

Not applicable
